# Antibacterial prescription and the associated factors among outpatients diagnosed with respiratory tract infections in Mbarara Municipality, Uganda

**DOI:** 10.1186/s12890-021-01739-5

**Published:** 2021-11-15

**Authors:** Timothy Eria Muwanguzi, Tadele Mekuriya Yadesa, Amon Ganafa Agaba

**Affiliations:** 1 Department of Pharmacy, Faculty of Medicine, Mbarara University of Science and Technology, Mbarara, Uganda; 2 Mubende Regional Referral Hospital, Mubende, Uganda; 3 World Bank, ACE II, Pharmacy Biotechnology and Traditional Medicine Center, Mbarara University of Science and Technology, Mbarara, Uganda; 4 Department of Pharmacy, Ambo University, Ambo, Ethiopia; 5 Department of Pharmacology, Faculty of Medicine, Mbarara University of Science and Technology, Mbarara, Uganda

## Abstract

**Background:**

Respiratory tract infections (RTI) are the second most frequent diagnosis after Malaria amongst Outpatients in Uganda. Majority are Non pneumonia cough and flu which are self-limiting and often do not require antibacterials. However, antibiotics are continuously prescribed for these conditions and are a major contributor to antimicrobial resistance and wastage of health resources. Little is known about this problem in Uganda hence the impetus for the study.

**Objectives:**

To determine the antibacterial prescribing rate and associated factors among RTI outpatients in Mbarara municipality

**Methodology:**

This was a retrospective cross-sectional study on records of RTI outpatients from 1st April 2019 to 31st March 2020 (prior to the novel corona virus disease pandemic) in four selected public health facilities within Mbarara municipality. A pretested data caption tool was used to capture prescribing patterns using WHO/INRUD prescribing indicators. We used logistic regression to determine factors associated to antibacterial prescribing.

**Results:**

A total of 780 encounters were studied with adults (18-59 years) forming the largest proportion of age categories at (337, 43.15%) and more females (444, 56.85%) than men (337, 43.15%). The antibacterial prescribing rate was 77.6% (606) with Amoxicillin the most prescribed 80.4% (503). The prescribing pattern showed an average of 2.47 (sd 0.72) drugs per encounter and the percentage of encounters with injection at 1.5% (24). Drugs prescribed by generic (1557, 79%) and drugs prescribed from essential medicine list (1650, 84%) both not conforming to WHO/INRUD standard; an indicator of possible irrational prescribing. Female gender (adjusted odds ratio [aOR] = 1.51, 95% confidence interval [CI]: (1.06–2.16); 18–59 years age group (aOR = 1.66, 95% CI: 1.09–2.33) and Individuals prescribed at least three drugs were significantly more likely to have an antibacterial prescribed (aOR= 2.72, 95% CI: 1.86–3.98).

**Conclusion:**

The study found a high antibacterial prescribing rate especially among patients with URTI, polypharmacy and non-conformity to both essential medicine list and generic name prescribing. This prescribing pattern does not comply with rational drug use policy and needs to be addressed through antimicrobial stewardship interventions, prescriber education on rational drug use and carrying out more research to determine the appropriateness of antibacterial prescribed.

## Introduction

Antibacterial misuse is a major contributor to antimicrobial resistance - an emerging global threat to the achievements of modern medicine [1]. Globally, around 50% of antibiotic prescriptions are incorrect [2] and inappropriate use of antibacterials in upper respiratory tract infections (URTI) have been reported [3]. In Uganda the Annual health sector report of 2018/2019 showed that Upper respiratory tract infections are the second most common causes of Hospital attendance at 10.6% just after Malaria at 12.5% [4].

In majority of these cases the Uganda clinical guidelines recommend supportive treatment due to their viral etiology. However a study in Mubende Regional Referral Hospital reported that over 81% of outpatients with upper respiratory infection received an antibiotic [5]. Furthermore, high rate of antibacterial use among children with acute respiratory tract infection was reported in northern Uganda at 60.2% in a house hold survey study [6]. Another household study in Kampala region among children reported 53.5% of the acute respiratory as inappropriately managed [7]. These findings point at possible misuse of antibacterial in Uganda but no study has been done to find out the burden in southwestern part of the country. Studies on antibacterial use amongst acute respiratory tract infection have reported high prevalence of misuse of antibiotics in Uganda at over 50% [6, 7].

Misuse of antibacterials is associated with increased morbidity, mortality, wastage of health resources and bacterial resistance. It is estimated that antimicrobial resistant infections cause about 50,000 deaths a year in Europe and the United states of America (USA) alone, perhaps aggregating to several hundred thousand when other countries are added [8]. Apart from the mortality associated with it, undue use of antibiotics increases medical cost in healthcare facilities [9]. The growing burdens associated with misuse of antibiotics are extremely worrying and the World Health Organization (WHO) assembly adopted a global action plan to fight antimicrobial resistance, in which antibiotic surveillance was emphasized. Examining prescriptions for rational drug use is a widely accepted strategy by WHO through which set indicators can assist in achieving the above goal [10].

Several studies on prescribing pattern have been carried out in other parts of Africa. In Nigeria the study noted poly pharmacy and non-adherence to essential medicine list [11]. Further still in eastern Ghana’s public health facilities antibiotic use was observed to be high compared to levels of developing countries throughout the world [12]. In Cameroun antibiotics were wrongly used in management of indications such as Uncomplicated Malaria that do not require antibiotic intervention in their standard management guides [13]

In Uganda the Annual health sector report of 2018/2019 showed that Upper respiratory tract infections (URTI) are the second most common causes of Hospital attendance at 10.6% just after Malaria at 12.5% [4]. In majority of these cases the Uganda clinical guidelines recommends supportive treatment due to their viral aetiology. However a study in Mubende Regional Referral Hospital reported that over 81% of outpatients with upper respiratory infection received an antibiotic [5]. Furthermore, high rate of antibacterial use among children affected by acute respiratory tract infection was reported in northern Uganda at 60.2% [6]. A similar study in Kampala region reported 53.5% of the acute respiratory cases as inappropriately managed [7]. These findings point at possible misuse of antibacterials in Uganda.

Respiratory tract infections are among the most prevalent cases in outpatient department and thus contribute highest to prescriptions that lead to misuse of antibiotics [7]. Unfortunately, there is a limited insight into the antibacterial use and prescribing pattern among outpatients diagnosed with respiratory tract infections in the Mbarara municipality, and as such this study aims to assess the antibacterial prescribing rate, RTI prescription pattern and the factors associated to antibiotic exposure.

## Methodology

### Study design and setting

This was a retrospective cross-sectional analysis study of medical records on RTI outpatients in four randomly selected health facilities of different service level in Mbarara Municipality, Southwestern Uganda. The Municipality has thirteen government health facilities at different health care levels [14]. According to the 2014 National Population and Housing Census, the municipality had a population of 195,318 people (Statistics [15]. The selected health facilities included (i) Mbarara Regional Referral Hospital (MRRH) (ii) Mbarara Municipal Health Centre IV, (iii) Kakoba Health Centre III, and (iv) Nyamityobora Health Centre II.

### Study population

The study population involved all outpatients diagnosed with RTI at selected Health facilities in Mbarara Municipality from 1st April 2019 to 31st March 2020 (prior to the Corona virus disease-COVID-19 pandemic). We excluded medical records missing vital information such as age and gender.

### Sample size

Based on the WHO recommendation on how to investigate drug use in Health facilities i.e., at least 600 encounters to be included in a cross-sectional survey of medication use, and reviewing of at least 100 encounters per Health facility to describe or compare drug use by individual facilities [16].

We used a sample size of 780 encounters of RTI because it allowed the lowest facility to have at least 100 encounters reviewed as recommended by WHO [17].

### Facility sample size

The individual facility sample size was calculated to put into consideration the difference in patient load. The regional referral had a highest patient load as such a higher sample was taken compared to other health facilities.

$$= \frac{Average\; monthly\; for\; facility}{{Monthly\; total\; of\; all \;facilities}} \times study\; sample \;size$$

MRRH

= (392/1030) 780 = 297

Mbarara Municipal Health Centre IV

= (274/1030) 780 = 208

Kakoba Health Centre III

= (230/1030) 780 = 175

Nyamityobora Health Centre II

= (133/1030) 780 = 101

### Sampling technique

Multi-stage sampling at the facility and the medical record was used. The facilities were selected by level of service they included Mbarara Regional Referral Hospital and Mbarara Municipal Health Centre IV are purposively selected being the only ones at that level.

A simple random sampling technique was used to select Nyamityobora Health Centre II (7 health Centre II available) and Kakoba Health Centre III (4 health Centre III available).

Specific medical records were selected using a systematic random sampling method using the medical record number as a reference for the sampling frame

Sampling interval

$$SAMPLE\; INTERVAL = \frac{Annual \;Total \;RTI\; patients \;in \;facility}{{weighted\; sample \;size\; of\; the\; facility}}$$

MRRH = 4104/297 = 13

Health Centre IV = 2894/208 =13

Kakoba Health Centre III = 2052/175= 12

Health Centre II =1596/101= 15

The first record was chosen at random from the first sample frame by computer generated number and then every nth medical record encounter from this initial number was included in the study.

### Data collection

Data was collected by the research team from hard copy medical records using a pretested web based google data extraction form with multiple checks to reduce entry errors. The form captured information from the out patients register which included: age, gender, month of treatment for RTI, route of administration, name of antibiotic prescribed, diagnosis, comorbidities, attendance (new vs old), malaria laboratory test, other medications, level of health facility, use of a generic name and if drug prescribed is on the essential drug list.

The clinician defined diagnosis was reclassified into three categories basing on the anatomical location i.e., upper respiratory tract infection, lower respiratory infection, and unspecified respiratory tract infection. Pharmacological classification was used to categorize the antibacterial drug prescribed.

### Ethical consideration

The study was conducted in accordance with the Declaration of Helsinki. The study received ethics approval from research ethics committee of Mbarara University of Science and Technology (Approval Number: MUSTREC 09/7). Permission to collect data was sought from the district health officer and the head of each facility. Patient name or other identifiers were not captured.

### Data analysis

A Microsoft excel sheet of the entered data was cleaned and data was then imported into Stata package 16.0 for statistical analysis.

Descriptive statistics was used to summarize the characteristics of patients with respiratory tract infection and presented as a percentage in each category. Logistic regression was used in the analysis to determine the association between the primary outcome (antibiotic prescribing) and the explanatory factors (age, gender, comorbidities, the period of visit, the total number of drugs prescribed, Upper respiratory tract infection diagnosis, health facility level, and laboratory test). Results of the analysis were presented as Odds Ratio (OR) at a confidence interval of 95% with *P*-value ˂ 0.05 considered significant.

## Results

### Characteristics of the RTI encounters

The study was conducted on 781 RTI outpatient prescription records in four selected health facilities within Mbarara municipality. The adults (18–59 years) formed the largest proportion of age categories at (337, 43.15%) and majority of the encounters were females (444, 56.85%). Unspecified URTI diagnosis was the most encountered (177, 22.6%) followed by unspecified RTI (145, 18.6%). For details see Table 1.

**Table 1:** Characteristics of prescriptions records of RTI Outpatients from April 2019 to March 2020 in Mbarara municipality

Variable	Category of variable	n (%)
Health Facility	MRRH	297 (38.03)
	Municipal	208 (26.63)
	Kakoba	175 (22.41)
	Nyamityobora	101 (12.93)
Age	Young child (0 – 4)	253 (32.39)
	Child (5 – 17)	173 (22.15)
	Adult (18 -59)	337 (43.15)
	Elderly (>60)	18 (2.30)
Sex	Female	444 (56.85)
	Male	337 (43.15)
Diagnosis (Clinician defined)	URTI unspecified	177 (22.66)
	Common cold	136 (17.41)
	Rhinitis	10 (1.28)
	Sinusitis	7 (0.9)
	Laryngitis	2 (0.26)
	Pharyngitis	11 (1.41)
	Tonsillitis	60 (7.68)
	Bronchitis	95 (12.16)
	Pneumonia	122 (15.62)
	Respiratory tract allergy	16 (2.05)
	RTI unspecified	145 (18.57)
*RTI diagnosis categorized	URTI	403 (51.60)
	LRTI	217 (27.78)
	Unspecified	161 (20.61)
Attendance	New	773 (98.98)
	Re-attendance	8 (1.02)
Weather season	Dry	378 (48.4)
	Wet	403 (51.6)
Lab test done (Malaria)	No	573 (73.37)
	Yes	208 (26.63)
**Comorbidity/Secondary diagnosis	No	691 (88.48)
	Yes	90 (11.52)

^*^RTI categorized: Diagnosis is categorized by researcher in terms of anatomical location of the clinician defined condition. Anatomically, respiratory tract is divided into upper (organ outside thorax—nose, pharynx and larynx) and lower respiratory tract (organ within thorax - trachea, bronchi, bronchioles, alveolar duct and alveoli).

^**^Comorbidity/ Secondary diagnosis: Any other diagnosis recorded by the researcher in addition to respiratory tract indications.

### Antibacterial prescribing rate among RTI outpatients

The overall rate of prescribing antibacterials among RTI outpatients was (606, 77.59%). The facility with the highest rate was municipal council (HCIV) at (174, 83.7%) closely followed by Kakoba HCIII (142, 81.1%) and Regional referral at (213, 71.7%) had the least rate amongst the health facility. The age group with the highest antibacterial prescribing rate was the elderly (16, 88.89%) followed by adults (279, 82.79%). Female who received antibacterials were (359, 80.86%) compared to males (247, 73.29%). The antibacterial prescription rate among the different patient diagnosis was Pneumonia at (119, 97.54%), URTI unspecified (154, 87.01%), RTI unspecified (123, 84.83%), Bronchitis (79, 83.16%), Pharyngitis (11, 100%), Allergy of Respiratory tract (8, 50%) and tonsillitis (51, 85%) (Table 2).

**Table 2:** Antibacterial prescribing rate across the different categories of RTI outpatients in Mbarara Municipality from April 2019 to March 2020

Variable class	Variable	n (%)	Yes= 606 (77.6%)	No = 175 (22.4%)	X^2^, (*p*-value)
Health facility	Hospital	297 (38.1)	213 (71.7)	84 (28.3)	11.67 (**0.009)**
	Health center IV	208 (26.6)	174 (83.7)	34 (16.3)	
	Health center III	175 (22.4)	142 (81.1)	33 (18.9)	
	Health center II	101 (12.9)	77 (76.2)	24 (23.8)	
Age	Young child (0–4)	253 (32.4)	180 (71.5)	73 (28.5)	16.17 **(0.003)**
	Child (5–17)	173 (22.1)	131 (75.7)	42 (24.3)	
	Adult (18–59)	337 (43.2)	279 (82.8)	58 (17.2)	
	Elderly (>60)	18 (2.3)	16 (88.9)	2 (11.1)	
Sex	Female	444 (56.8)	359 (80.9)	85 (19.1)	6.30 **(0.012)**
	Male	337 (43.2)	247 (73.3)	90 (26.7)	
RTI diagnosis categorised*	URTI	403 (51.6)	277 (68.7)	126 (31.2)	39.92 (<**0.0001**)
	LRTI	217 (27.8)	198 (91.2)	19 (8.8)	
	Unspecified	161 (20.6)	131 (81.4)	30 (18.6)	
Attendance	New	773 (99.0)	601 (77.7)	172 (22.3)	1.06 (0.303)
	Re-attendance	8 (1.0)	5 (62.5)	3 (37.5)	
Weather season	Dry	378 (48.4)	302 (76.9)	76 (20.1)	2.23 (0.135)
	Wet	403 (51.6)	304 (75.4)	99 (24.6)	
Lab test done (Malaria)	No	573 (73.4)	442(77.1)	131 (22.9)	0.26 (0.613)
	Yes	208 (26.6)	44(21.1)	164 (78.9)	
Comorbidity/ Secondary diagnosis	No	691 (88.5)	536 (77.6)	155 (22.4)	0.002 (0.964)
	Yes	90 (11.5)	70 (77.8)	20 (22.2)	

^*^RTI categorized: Diagnosis is re-categorized by anatomical location that is affected, URTI = upper respiratory tract infections, LRTI = lower respiratory tract infections

### Antibacterial prescribing rate per categorised diagnosis

**Fig. 1:**
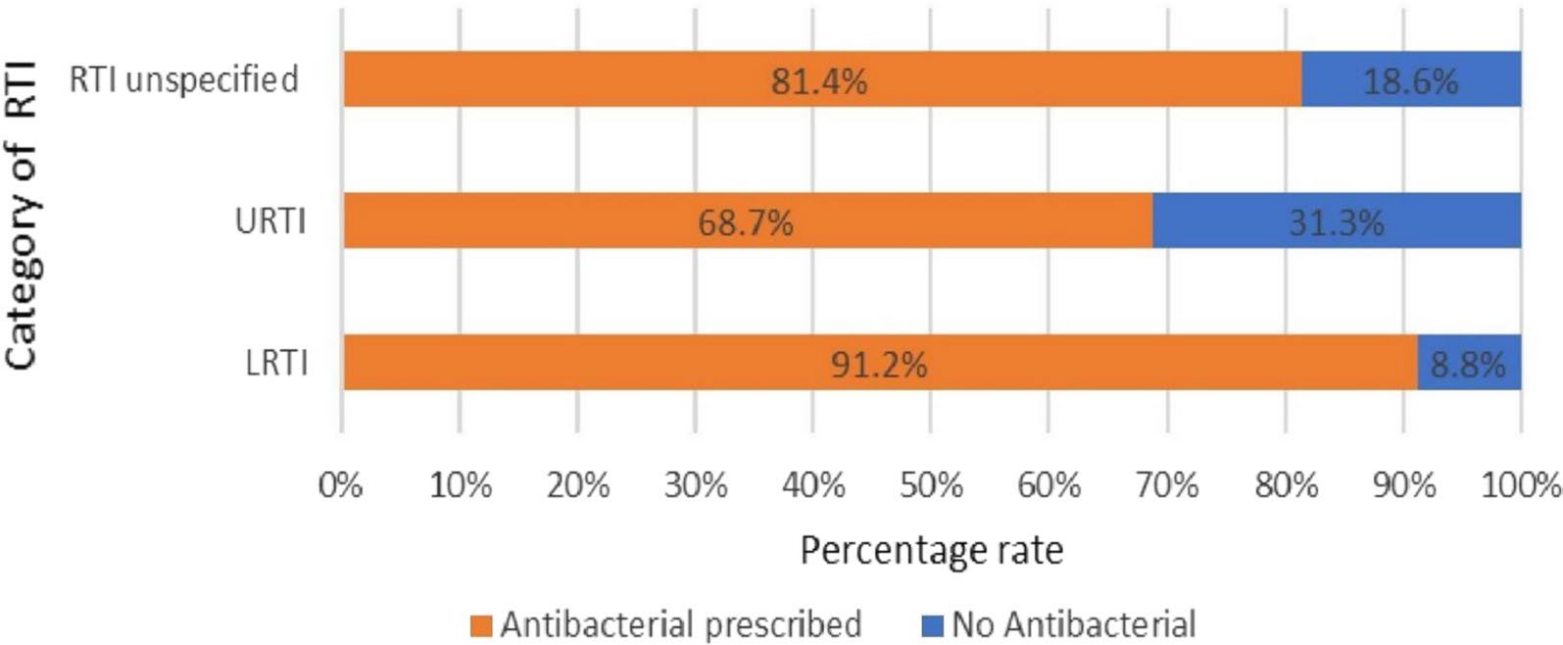
Prevalence of antibacterial prescribing rate per categorized diagnosis among outpatients in Mbarara municipality from April 2019 to March 2020

The antibacterial prescribing rate per categorised diagnosis was 68.7% in patients with Upper respiratory tract infection, and 81.4% with unspecified respiratory tract infection and highest among Lower respiratory tract infection 91.2% (Fig. 1).

### Prevalence of prescribed antibacterial by classes

**Fig. 2:**
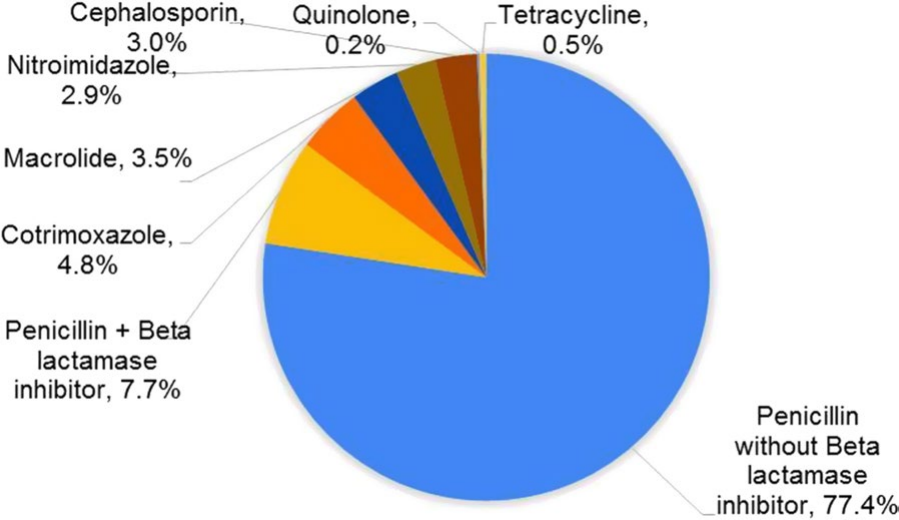
shows the proportion of antibacterial class prescribed among RTI outpatients in Mbarara Municipality from April 2019 to March 2020

The penicillins without beta lactamase inhibitor (484, 77.4%) formed the highest proportion of prescribed antibacterials followed by penicillins with beta-lactamase inhibitors (48, 7.7%). Cotrimoxazole at (30, 4.8%), macrolides at (22, 3.5%), tetracycline and quinolone the least prescribed at (3, 0.5%) (1, 0.2%) respectively (Fig. 2)

### Proportion of specific antibacterial prescribed

Amoxicillin was the most prescribed antibacterial (455, 72.8%) followed by amoxicillin with clavulanic acid at (48, 7.68%) then Cotrimoxazole (30, 4.80%). Benzathine (1, 0.16%), levofloxacin (1, 0.16%) were the least prescribed antibacterials (Table 3).

**Table 3:** Proportion of specific antibacterials prescribed for RTI out patients in Mbarara Municipality from April 2019 to March 2020

Class	Drug name	Frequency	%
Penicillin	Amoxicillin	455	72.80
	Amoxicillin +clavulanic acid	48	7.68
	Ampicillin+cloxacillin	10	1.60
	Benzyl penicillin	4	0.64
	Benzathine	1	0.16
	Flucloxacillin +amoxicillin	11	1.76
	Phenoxymethylpenicillin	3	0.48
Cotrimoxazole	Cotrimoxazole	30	4.80
Macrolide	Azithromycin	14	2.24
	Erythromycin	8	1.28
Nitro imidazole	Metronidazole	18	2.88
Cephalosporin	Cephalexin	7	1.12
	Cefixime	7	1.12
	Ceftriaxone	5	0.80
Quinolone	Levofloxacin	1	0.16
Tetracycline	Doxycycline	3	0.48

### Respiratory tract infection prescription pattern

The average number of drugs per encounter was 2.47 for all facilities, the highest being HC IV at 2.67 drugs, and regional referral at 2.38 drugs. The average number of prescriptions with antibiotics prescribed (77.59%) with the highest being HC IV (83.65%), Percentage of encounters with injections was 1.54% with HCII reporting no use of injections. Percentage of drugs prescribed by generic was 80.84% with MRRH reporting the lowest 57.49% and HCIII the highest conformity to generic prescribing at 97.37%. The proportion of prescribed drugs from Essential drug list was 85.67% (Table 4).

**Table 4:** Drug prescribing evaluation using WHO/INRUD indicators among RTI outpatients in Mbarara Municipality for a period from April 2019 to March 2020

Health center/indicator	WHO Standard	All facilities	Regional Referral	Municipal Health Centre IV	Kakoba Health Centre III	Nyamityobora Health Centre II
Total number of drugs prescribed	*	1926	708	555	418	245
Total number of encounter	*	781	297	208	175	101
Number of injections prescribed	*	12	6	5	1	0
No of generic drugs prescribed	*	1557	407	510	407	233
Total Number of drugs prescribed from EML	*	1650	490	515	408	237
Average number of drugs prescribed per encounter	<2	2.47				
	2.38	2.67	2.39	2.42		
Percentage of encounters with an antibacterial prescribed	<30^a^	77.59	71.72	83.65	81.14	76.24
Percentage of encounters with an injection prescribed	<10	1.52	2.02	2.4	0.57	0
Percentage of drugs prescribed by generic	100	80.84	57.49	91.82	97.37	95.10
Percentage of drugs prescribed from EML	100	85.67	69.21	92.79	97.61	96.73

^a^WHO recommendation of Average encounters with Antibacterials is on general outpatients, not specific infection such as RTI

^*^No standard set by WHO

### Factors associated with antibacterial prescribing

#### Bivariate logistic regression analysis

The bivariate logistic regression showed that attending HC IV [COR = 2.02, 95% CI: 1.29–3.15, *p*-value 0.002] and HC III [COR 1.7, 95% CI: 1.07–2.67, *p*-value 0.023], being an adult (18–59 years) [COR = 1.95, 95% CI: 1.32–1.89], being female [COR = 1.54, 95% CI: 1.10–2.16], having a diagnosis LRTI [COR = 15.82, 95% CI: 4.95–50.58], unspecified RTI diagnosis [COR = 1.74, 95% CI: 1.12–2.71, *p* value 0.014], and having a prescription with three or more drugs [COR = 2.52, 95% CI: 1.91–3.33], were all significantly associated with antibacterial prescribing (Table 5).

**Table 5:** Bivariate analysis of factors associated to antibacterial prescribing among RTI outpatients in Mbarara municipality from April 2019 to March 2020

Variable	Variable category	Antibiotic		COR (95% CI)	*P* value
		Yes	No		
Health Facility*	MRRH (Referral)	213 (71.72)	84 (28.28)	1	
	Municipal (HCIV)	174 (83.65)	34 (16.34)	2.02 (1.29–3.15)	**0.002**
	Kakoba (HC III)	142 (81.14)	33 (18.86)	1.7 (1.07–2.67)	**0.023**
	Nyamityobora (HC II)	77 (76.24)	24 (23.76)	1.26 (0.75–2.13)	0.378
Season	Dry	302 (76.89)	76 (20.11)	1	
	Wet	304 (75.43)	99 (24.57)	0.77 (0.55–1.08)	0.136
Age*	Young child (0 – 4)	180 (71.15)	73 (28.5)	1.	
	Adolescent (5 – 17)	131 (75.72)	42 (24.28)	1.26 (0.81–1.97)	0.297
	Adult (18 -59)	279 (82.79)	58 (17.21)	1.95 (1.32–2.89)	**0.001**
	Elderly (>60)	16 (88.89)	2 (11.11)	3.24 (0.73–14.47)	0.123
Sex*	Male	247 (73.29)	90 (26.71)	1	
	Female	359 (80.86)	85 (19.14)	1.54 (1.10–2.16)	**0.012**
Lab test done	No	442 (77.14)	131 (22.86)	1	
	Yes	44 (21.15)	164 (78.85)	1.10 (0.75–1.62)	0.613
Attendance	New	601 (77.75)	172 (22.25)	1	
	Re-attendance	5 (62.5)	3 (37.5)	0.48 (0.11–2.02)	0.314
RTI diagnosis categorised*	URTI	277 (68.73)	126 (31.27)	1	
	LRTI	198 (91.24)	19 (8.76)	4.74 (2.83–7.93)	**<0.0001**
	Unspecified	131 (81.37)	30 (18.63)	1.74 (1.12–2.71)	**0.014**
Comorbidity	No	536 (77.57)	155 (22.43)	1	
	Yes	70 (77.78)	20 (22.22)	1.01 (0.60–1.72)	0.964
Total number of drugs*	2 drugs and below	302 (70.07)	129 (29.93)	1	
	3 drugs and above	304 (86.86)	46 (13.14)	2.82 (1.95–4.10)	**<0.0001**

*COR* crude odd ratio

^*^Exported to multivariate analysis model if *P* value was below 0.05

Bold: statistically significant

#### Multivariate logistic regression analysis

Multivariate analysis showed that female gender [aOR = 1.5, 95% CI: 1.07–2.15 *p* = 0.021], adult age group [aOR = 1.66, 95% CI: 1.07–2.15, *p* = 0.018] as well as having a prescription with three or more drugs [aOR = 2.72, 95% CI: 1.07–2.15, p= 0.0001] were more likely to be prescribed antibiotics. (Table 6).

**Table 6:** Multivariate analysis of factors associated with antibacterial prescribing among RTI outpatients in Mbarara municipality from April 2019 to March 2020

Variable	Categories of variable	**Antibiotic prescription**	**aOR (95% CI)**	**(P value)**	
		**Yes**	**No**		
Age	Young child (0 – 4)	180 (71.15)	73 (28.5)	1	
	Adolescent (5 – 17)	131 (75.72)	42 (24.28)	1.21 (0.76–1.90)	0.422
	Adult (18 -59)	279 (82.79)	58 (17.21)	1.66 (1.09–2.53)	**0.018**
	Elderly (>60)	16 (88.89)	2 (11.11)	2.48 (0.53–11.47)	0.246
Sex	Male	247 (73.29)	90 (26.71)	1	
	Female	359 (80.86)	85 (19.14)	1.51 (1.07–2.15)	**0.021**
Total number of drugs prescribed	2 drugs and below	302 (70.07)	129 (29.93)	1	
	3 drugs and above	304 (86.86)	46 (13.14)	2.72 (1.86–3.98)	**<0.0001**
Health facility level	Referral Hospital	213 (71.72)	84 (28.28)	1	
	HC IV	174 (83.65)	34 (16.34)	1.45 (0.89–2.36)	0.14
	HC III	142 (81.14)	33 (18.86)	1.54 (0.96–2.47)	0.075
	HC II	77 (76.24)	24 (23.76)	1.02 (0.59–1.78)	0.933
RTI diagnosis category	URTI	356 (71.49)	142 (28.51)	1	
	LRTI	119 (97.54)	3 (2.46)	0.37 (0.07–1.89)	0.232
	Unspecified	131 (81.37)	30 (18.63)	1.25 (0.61–2.56)	0.537

*aOR* adjusted odd ratio

## Discussion

Results show that the antibacterial prescribing rate among outpatients with RTI Mbarara municipality is 77.6%. They further show that 68.7% of all URTI encounters received antibacterials and beta lactams as the most prescribed antibacterials.

The prescribing rate means that approximately eight out of ten patients with a RTI were prescribed an antibacterial drug. This is inappropriately high given that only 217 (27.7%) of the patients were diagnosed with LRTI which mainly require antibacterials [5]**.** This Prescribing rate (77.6%) is higher than 60.2% reported by a cross sectional survey conducted in children under five years in Gulu, Northern Uganda [6] but lower compared to 92.7% from a previous study in Mubende regional referral Hospital of Uganda [5]. The latter study in Mubende had similar methodology as this study but difference in results could be due to the prescribing pattern of the prescribers in these two different regions.

The URTI category which included common cold, laryngitis, rhinitis, pharyngitis, rhinitis had close to 68.7% of these encounters receiving antibacterial drugs, which in most cases is inappropriate. This misuse of antibacterials as noted in numerous studies [18, 19] is contrary to Uganda clinical guidelines recommendations of supportive treatment. A similar study in Kenya by Momanyi in Rift valley Hospital [20], we reported beta-lactam as the most frequently prescribed antibacterials class at 88.2%. Amoxicillin was the most prescribed individual antibacterial drug at 72.8% and this is because it is the recommended drug of choice in empiric treatment of LRTI but its usage in 91% of encounters with URTI diagnosis without a secondary diagnosis points towards inappropriate use

The measure of the degree of polypharmacy (the practice of the administration/ of multiple medications at the same time) showed that the average number of drugs per encounter (2.5 drugs) was closely similar at all the different health facility levels but is slightly above the recommended WHO standard of below two drugs [16]. Higher degree of polypharmacy was also noted in a study in Ghana and Nigeria [11, 12] reported at an average of 4 drugs for the general outpatients which was higher than the results in this study. A different study by Yimenu in Nigeria reported lower average number of drugs per prescription of 1.6 drugs [21]. These differences are probably due to variations in the prescribers’ prescribing habits. Despite polypharmacy presenting several challenges some scholars are against the use of numerical thresholds like average number of drugs per script in measuring polypharmacy because they do not put into consideration the clinical context such as multimorbid individuals [22, 23].

Encounters with injection rates were within the recommended range of <10% [16]. The use of injections among RTI outpatients seemed to increase as we go up the level of health facility with health Centre II having no injection whereas HC IV and regional referral had percentages of 2.4% and 2% respectively. This was likely because the lower health facilities do not stock these injection products and also the referral system allows more complicated/severe cases which may need parenteral route to be handled by higher level health facilities. Findings at our lower healthcare centers were similar to a study carried out in India [24].

The average percentage of drugs prescribed by generic was 80.8% which was below the 100% recommendation by WHO [16]. Moreover, there was observed a high difference among the lower health facilities and Regional referral hospital on the tendency to prescribe by generic name, with percentages of 94%, 97% and 92% for HCII, HCIII and HCIV respectively as compared to 55% for the Regional referral hospital. The difference could be attributed to medical marketing which emphasizes brand prescribing focusing on high level hospitals based in bigger towns [25]. This generic name prescription rate of 55% by the regional referral hospital is comparable to 62.5% prevalence in a referral hospital in Kenya at 62.5% [20]. This finding by the current study is against the recommendations that prescribing in generic show that it lowers costs because cheaper generics can be used, reduces confusion among health workers and minimizes influence of marketing on prescribing [25].

The results further showed that the percentage of drugs prescribed from essential drug list was 84%, lower-level health facilities, HCII, HCIII and HCIV showed 96%, 97%, 93% adherence to the essential medicine list respectively whereas MRRH showed 62%, though WHO recommendation is 100%. This showed a small non adherence to the essential medicine list at lower health facilities and a major difference by prescribers in the referral hospital which may be explained by drug marketing that which influences the choice of drug a prescriber chooses [25], and possibly lack of supervision of the prescribers on drug policies. Several studies have reported results short of the essential medicine prescribing target in Africa [12], in China [26] while some show compliance in Cameroun [13].

The study identified female gender, adult age (18–59) and prescribing 3 or more drugs as independent associated factors to the antibacterial prescribing. Accordingly, female patients were about 1.5 times more likely to be prescribed antibiotic compared to males and adults were about 1.66 times more likely to be prescribed an antibacterial. Likewise, those who were prescribed 3 or more drugs were about 2.7 times more likely to be prescribed an antibacterial compared to those who were prescribed 2 or less drugs.

Similarly, several other previous studies also showed that being female was independently associated with antibacterial prescribing [13, 27, 28]. This can be explained by the fact that females have higher health care seeking behavior compared to males,thus interact more with prescribers and as such possibly demand for the antibacterials [29].However, this gender association was contrary to findings by a study in Ethiopia [30] and a study in Bangladesh [31] where males were more likely to receive antibiotics. Several reasons linked to this gender disharmony in antibacterial prescribing include the difference in social and behavioral factors among males and females.

On the other hand, the higher propensity for the adult age group (18-59 years to be prescribed antibacterials compared to the young (below 5 years) may be attributed to several factors which include demand for antibiotics by the patients or pressure by the prescriber not to disappoint and as such prescribe antibacterials several studies have elucidated this association with the antibacterial prescribing [32–34].

The current finding that the higher the number of drugs prescribed, the higher the chances of getting an antibacterial prescribed, is also comparable with findings of previous studies [35]. This can be explained by the fact that if a prescriber is prone to overprescribing then it is more likely that antibacterials shall be prescribed when they are not necessary

### Strengths and limitation

The data collected was for one year and this catered for the possible seasonal variations in antibacterial prescribing, this study period is in agreement with WHO recommendations. This was a retrospective study that looked at outpatient records and this was intended to avoid Hawthorn effects (clinician awareness that they are being observed) however this led to many factors associated with antibacterial prescription such as signs and symptoms, patient load, previous treatment, acuteness of the illness, patient follow up and prescriber based factors, not to be studied due to lack of this data in the records. The total number of four health facilities studied is small for drug use evaluation studies (due to financial and time constraints) and thus makes it impossible to generalize the results to a region or country. However the results shade light on the possible misuse given that other scholars have highlighted it.

## Conclusion

The antibacterial prescribing rate in Mbarara municipality was high at 77%, yet LRTI encounters formed 27.78% of all encounters, this discrepancy indicates unnecessary antibiotic exposure hence antibiotic irrational use. RTI Outpatients were averagely prescribed 2.47 drugs per prescription which is slightly above the WHO recommended standard. There is non-conformity to the national drugs policy as evidenced by the percentage of drugs prescribed from the national essential medicine list at 84% and the tendency to prescribe by generic name at 80.8%. This non adherence was more reported at the regional referral Hospital compared to the other health facilities. The study also showed that being female, age group (18–59 years) and total drugs prescribed (at least three) were associated with antibacterial prescribing.

## Data Availability

All the data supporting the conclusions of this article is included within this manuscript.

## Abbreviations

AMR: Anti-Microbial Resistance
EML: Essential Medicine List
HC: Health Centre
IDI: Infectious Disease Institute
INRUD: International Network for Rational Use of Drugs
LRTI: Lower respiratory tract infection
MRRH: Mbarara Regional Referral Hospital
MOH: Ministry of Health
NGO: Non-Governmental Organisation
OPD: Out Patients Department
RTI: Respiratory Tract Infection
TB: Tuberculosis
VIF: Variance inflation factor
URTI: Upper Respiratory Tract Infection
USD: United States Dollar
WHO: World Health Organization
